# Inpatient Discharge Letters

**DOI:** 10.1192/bjo.2026.11728

**Published:** 2026-06-30

**Authors:** Ezzat Elesseily, Jennifer Salmons

**Affiliations:** EPUT, Chelmsford, United Kingdom

## Abstract

**Aims:**

Essex partnership university Trust (EPUT) policy on Discharge and Transfer Clinical Guidelines (CG24) aims to provide a clear pathway for the transfer and discharge of all patients of EPUT from and within Mental Health, Learning Disability, Secure Services and Community Health Services. It also aims to ensure that a patient’s transition between areas of EPUT services and transfer outside of EPUT services is carried out timely, effectively and safely.

The aim of audit is to to evaluate if EPUT Policy on Discharge and Transfer Clinical guidelines CG24 was being followed in an inclusive inpatient setting.

**Methods:**

Data was collected retrospectively on all discharges from 6 acute inpatient wards within the Mid locality of EPUT; in the Linden Centre, Chelmsford.Inpatient wards that were included were General Adult (Galleywood, Finchingfield, Topaz), Older Adult (Ruby), Perinatal Specialist (Rainbow) and *Psychiatric* Intensive Care Unit (Christopher Unit) using a custom built audit tool. All discharges in a 3-month period (from June 2025 to August 2025) were included. This information was gathered from the electronic record (PARIS) with sample size of 91 discharges.

**Results:**

1. Copy of the brief discharge summary given to patient: 100%
2. Copy of detailed discharge summary scanned onto electronic medical records: 98%.
3. GP to receive brief summary about admission, treatment required and medications within 24 hours of patient leaving the ward: 86%
4. Copy of brief discharge summary scanned onto electronic medical records: 86%
5. GP to receive detailed discharge summary within 5 working days of patient leaving the ward: 81%
6. Copy of the more detailed discharge summary sent to patient: 0%

**Conclusion:**

This re-audit has highlighted good compliance with providing the brief summary to the patient directly and scanning detailed discharge summary onto electronic medical records. There is a slight decline in sending both the brief summary to the GP within 24 hours, and with sending the detailed summary to the GP within 5 days following discharge compared to previous audit cycle with 81 discharges (data collected February 2024 – April 2024).

**Areas of Good Practice:**

Wards were generally compliant at uploading detailed discharge summaries to the EPR (98%).

Nurses continued to provide patients with a copy of brief discharge summary. (100%)

All wards apart from Rainbow (Mother and baby unit) are using E discharge summary.

**Areas for Improvement:**

There was slight decline in sharing of the brief and detailed discharge summaries with the GP, and poor compliance in sharing detailed discharge summaries with patients.

